# Getting Straight on What’s Flushed: “Sewage Epidemiology” Measures Community Drug Consumption

**Published:** 2008-08

**Authors:** Tanya Tillett

Active pharmaceutical agents and other chemicals in sewage pose a considerable concern when one considers the potential for inadvertent exposures through treated water. On the flip side, wastewater can also provide a wealth of economical and accessible epidemiologic data on common drug products consumed and excreted into community sewage systems. Now researchers have successfully tested a new “sewage epidemiology” analysis strategy to obtain near real-time information on community usage rates of drugs, allowing trends and patterns to be promptly monitored **[*EHP* 116:1027–1032; Zuccato et al.]**.

Many drug usage studies focus on prevalence data—reported use rates based on the integration of population surveys with medical records, drug production and seizure rates, and crime statistics. But obtaining information through these channels can often be time-consuming and the accuracy questionable, as the data are based partly on self-reported use. Using a novel approach first proposed in 2001, the researchers in the current study gathered data from sewage treatment plants in Milan (Italy), Lugano (Switzerland), and London (England) to obtain information on community-wide consumption of cocaine, heroin, cannabis, and amphetamines.

The investigators collected composite samples of untreated wastewater from major sewage treatment plants in each of the cities every 20 minutes for 24 hours in a time-proportional mode and pooled the samples using a computer-controlled device. They also tested field data from a given treatment plant for reproducibility over time: samples were taken on consecutive days for 1 week on 3 different occasions in Milan and Lugano, and on 2 days at 2 major plants in London. Wastewater samples were analyzed by liquid chromatography–tandem mass spectrometry, which measured drug residues using a highly selective multiresidue assay.

This new testing method enabled the research team to accurately measure the drugs in wastewater samples using objective quantitative data—drug concentration, wastewater flow rate, and population size—and to acquire near real-time reporting of results because of the short 1- to 2-day completion time for mass spectrometric analysis of samples. The new approach also makes it possible to integrate wastewater monitoring data with other information on drug use to obtain more refined estimates of community consumption patterns and user profiles.

The authors note this approach has certain limitations, including lack of data regarding the number of drug users in a given community, but say that the results on overall consumption rates compare reasonably well with official prevalence-based figures. For the sake of accuracy, detailed information would be needed on the metabolism and kinetics of any compound for which the approach is used. Furthermore, it can be difficult to accurately characterize “typical” users of certain pharmaceuticals or drugs. However, the authors state that with further testing, the method could be used in future research to provide real-time epidemiologic data for application to other public health issues.

